# Nurses’ Use of Mobile Devices to Access Information in Health Care Environments in Australia: A Survey of Undergraduate Students

**DOI:** 10.2196/mhealth.3467

**Published:** 2014-12-10

**Authors:** Carey Mather, Elizabeth Cummings, Penny Allen

**Affiliations:** ^1^School of Health SciencesUniversity of TasmaniaLauncestonAustralia; ^2^Rural Clinical SchoolUniversity of TasmaniaBurnieAustralia

**Keywords:** undergraduate nurse, mobile, work integrated learning

## Abstract

**Background:**

The growth of digital technology has created challenges for safe and appropriate use of mobile or portable devices during work-integrated learning (WIL) in health care environments. Personal and professional use of technology has outpaced the development of policy or codes of practice for guiding its use at the workplace. There is a perceived risk that portable devices may distract from provision of patient or client care if used by health professionals or students during employment or WIL.

**Objective:**

This study aimed to identify differences in behavior of undergraduate nurses in accessing information, using a portable or mobile device, when undertaking WIL compared to other non-work situations.

**Methods:**

A validated online survey was administered to students while on placement in a range of health care settings in two Australian states.

**Results:**

There were 84 respondents, with 56% (n=47) reporting access to a mobile or portable device. Differences in use of a mobile device away from, compared with during WIL, were observed for non-work related activities such as messaging (*P*<.001), social networking (*P*<.001), shopping on the Internet (*P*=.01), conducting personal business online (*P*=.01), and checking or sending non-work related texts or emails to co-workers (*P*=.04). Study-related activities were conducted more regularly away from the workplace and included accessing University sites for information (*P*=.03) and checking or sending study-related text messages or emails to friends or co-workers (*P*=.01). Students continued to access nursing, medical, professional development, and study-related information away from the workplace.

**Conclusions:**

Undergraduate nurses limit their access to non-work or non-patient centered information while undertaking WIL. Work-related mobile learning is being undertaken, *in situ*, by the next generation of nurses who expect easy access to mobile or portable devices at the workplace, to ensure safe and competent care is delivered to their patients.

## Introduction

The rapid evolution of digital technology in health care environments has created new challenges for learning and teaching (L&T). While increasing access to mobile or portable devices has enabled opportunities for promoting learning at the workplace in real-time [1-3], there is the risk that portable devices may distract from patient or client care if used by health professionals or students during employment or work-integrated learning (WIL) [4-6]. Undergraduate nurses undertake one third of their course in a range of health care settings. Experiential learning provides students with the opportunity to link theory with practice and augments learning, *in situ*. Previous studies have indicated that access to mobile or portable devices at point of care, may be cause for concern regarding patient or client safety [4-8], professional identity [9,10], or workforce development opportunities [11,12]. However, there is little research regarding the frequency of use or the type of information accessed on mobile devices by undergraduate nurses during WIL.

The reported use, effectiveness, and impact of eHealth and mobile devices internationally is similar to the Australian situation. Systematic reviews demonstrate that evidence is required to guide clinicians and develop frameworks for use in clinical environments [13,14]. The Australian National E-Health Strategy identified that a health workforce skilled in information communication technology (ICT) was a key area for driving change that could transform health care delivery [15]. Furthermore, the Workforce Development Strategy [16] emphasized the need for more creative and effective use of ICT and the need to improve the digital literacy of health professionals. Studies have found that although health profession students report ubiquitous use of computers, it is not translated into ICT competency [17,18]. Inclusion of ICT literacy development in all undergraduate nursing programs is a requirement of the Australian Nursing and Midwifery Council [19]. National strategies and registered health profession bodies contend that educational preparation of student nurses in ICT literacy at an individual level is critical for ensuring competency that is reflected at a systems level in health care environments.

Gray and colleagues [20] reviewed the implementation and effectiveness of clinical informatics education for future health professionals and concluded that a more sophisticated and scholarly approach to further pedagogical enquiry into clinical informatics education was required. Lindley and Fernando [5] asserted that curriculum content and L&T approaches at a systems level needed to improve preparation of students for their future careers.

The second global survey on eHealth [21] identified a range of challenges that mobile technology posed at individual, organization and systems levels. Due to the complexity of the health sector, integrating mobile technologies into routine health care practice at point of care has been slow [21]. There is a range of factors that impact at an individual, organization, and systems level [22-24]. Environmental factors include institution and organization governance, policy and ICT architecture, or infrastructure that prohibit or reduce access to mobile or portable devices at the workplace [7].

Previous studies have focused on the technology available rather than the learning afforded by its use [2]. Students can now engage and capture, in real-time moments they regard as significant for learning [25] which are then used to scaffold understanding or build knowledge [26,27]. Mobile learning (mLearning) in the workplace enables a student-centered approach whereby an effective and efficient response can be obtained as they arise [2]. Students can merge the nexus of theoretical learning, while developing skills to augment their learning by accessing web-based resources such as YouTube clips, text or images for medication management or nursing diagnosis information. Additionally, informal learning at point of care creates opportunity for patient-centered, participatory care that could improve health outcomes by enabling access to resources and individualized treatment plans [28,29]. Mobile learners have considerable control over their learning, they can share, store, re-purpose, and re-use objects and artifacts for use in discussion, reflection, or peer review later at a more suitable place or time [30,31]. While mLearning also offers opportunities to access expert advice or opinion on a global scale, there are challenges and risks associated with introducing L&T innovation into the workplace.

Perceived risks associated with using mobile devices in the workplace have been investigated. Potential distraction from patient care while using a mobile device *in situ*, is well documented [5-7,20,32]. However, the benefits of accessing L&T information by using mobile devices at the point of care have been less thoroughly researched. Mather and colleagues [33] found there were a number of human factors that reduced the capacity of clinical supervisors in effectively using mobile learning approaches during WIL. These include intrinsic and extrinsic motivations [34,35], social presence, peer disapproval [33], or infection control [36,37]. The need to further explore the limited implementation of mobile learning using mobile devices has emerged leading to this study. This paper reports on the results of an online survey administered to undergraduate nurses in a range of health care settings. The aims of the survey were: (1) to advance understanding of how mobile devices are used to access information at, and away, from the workplace; and (2) to determine differences in accessing information by students during WIL or away from the workplace.

## Methods

### Study Design

This cross-sectional study captured self-report of undergraduate nurses’ access to Internet or device-based resources, using a mobile or portable device at, and away, from the workplace. The study involved administration of a survey to undergraduate nurses, while they were in clinical practice during January 2014, at a range of health care settings in two Australian states.

### Ethical Approval

Minimum risk ethics for this research was approved by the University of Tasmania Human Research Ethics Committee, approval number H0013729. Consent was implied by completion of the survey.

### Participant Recruitment

Eligible participants were identified through consultation with lecturers from the University. All participants were undertaking WIL and were recruited via email. Two reminder request emails were sent at two week intervals following the initial request.

### Data Collection

Of the 22 survey items relating to utilization of mobile devices to access information, 15 were from a validated tool developed by McBride, LeVasseur and Li [6]. Professional experience placement (PEP) was the term used in the survey to describe WIL. ‘Away from PEP’ was defined as when the student was not undertaking placement as part of their studies and ‘During PEP’ meant the student was undertaking workplace learning or clinical placement hours in a health care setting as part of their study. Five-point Likert scale questions (Scale of 1-5: 1: Never, 2: Once per day, 3: 2-5 times per day, 4: >5 times per day, 5: Not applicable) were used to determine frequency of use when away from and while undertaking WIL.

### Data Analysis

The survey data were imported into IBM SPSS (Version 21) for analysis and frequencies were investigated. Chi-square tests were utilized to explore differences between those who had access to a mobile device and those who did not. Differences in responses to scales for ‘Away from PEP’ and ‘During PEP’ were explored using Wilcoxon signed ranks tests. All tests were two-sided and differences were accepted at *P*<.05 significance level.

## Results

### Participants

A total of 476 students undertaking WIL were offered the opportunity to participate in the online survey and 84 responded (18% response rate). There were 37 respondents (44%) who participated in WIL in New South Wales, and 38 (45%) in Tasmania. Of those respondents, 45 (54%) were in their first year of nursing study. Furthermore, 44 respondents (52%) undertook WIL at tertiary health care facilities and the remainders were dispersed at district hospitals or community-based facilities.

A filter question requiring access to a mobile or portable device (Do you have current access to a mobile technology device?) rendered 37 respondents ineligible to complete the second section of the questionnaire. Table 1 presents demographic information for all respondents and those who had access to a mobile device. No differences were found in access to mobile devices for gender (χ2_1_=0.0, *P*=1.0), ethnicity (χ2_1_=0.0, *P*=1.0) or geographic location (χ2_1_=0.8, *P*=.4). There were insufficient expected cell frequencies to establish associations for age group, level of education, and focus of health care organization. Additionally, there was no difference between the two groups when the categories were collapsed to investigate associations between access to a mobile device and type of WIL (tertiary or other health care) organizations. Final year students were more likely to have access to a mobile or portable device than first year students (n=23, 77% versus n=24, 53%, χ2_1_=4.2, *P*=.04).

**Table 1:** Demographic information of respondent access to a mobile or portable device.

Demographic descriptor		All respondents N=84^a^	Access to a portable or mobile device N=47^a^
**Gender**			
	Male	19 (23%)	14 (30%)
	Female	56 (67%)	33 (70%)
	Missing; non respondents	9 (11%)	0 (0%)
		
**Age**			
	<21	10 (12%)	4 (9%)
	21-30	22 (26%)	13 (28%)
	31-40	20 (24%)	15(32%)
	41-50	13 (15%)	8 (17%)
	>51	10 (12%)	7 (15%)
	Missing	9 (11%)	0 (0%)
		
**Language, other than English spoken at home**			
	Yes	24 (29%)	14 (30%)
	No	51 (61%)	33 (70%)
	Missing	9 (11%)	0 (0%)
		
**Level of education prior to this course**			
	Secondary	20 (24%)	9 (19%)
	Vocational certificate	21 (25%)	15 (32%)
	Undergraduate degree	26 (31%)	18 (38%)
	Post graduate	8 (10%)	5 (11%)
	Missing	9 (11%)	0 (0%)
		
**State where student undertook WIL**			
	NSW	37 (44%)	23 (49%)
	TAS	38 (45%)	24 (51%)
	Missing	9 (11%)	0 (0%)
		
**Year of study**			
	First year	45 (54%)	27 (57%)
	Final year	30 (36%)	20 (43%)
	Missing	9 (11%)	0 (0%)
		
**Focus of care of health organization**			
	Major hospital	44 (52%)	31 (66%)
	District hospital	11 (13%)	5 (11%)
	Primary care	5 (6%)	2 (4%)
	RACF	3 (4%)	2 (4%)
	Multipurpose	2 (2%)	1 (2%)
	GP	3 (4%)	3 (6%)
	Mental health	4 (5%)	2 (4%)
	Other	3 (4%)	1 (2%)
	Missing	9 (11%)	0 (0%)
		
**Access to portable or mobile device**			
	Yes	47 (56%)	
	No	12 (14%)	
	Missing	25 (30%)	

^a^May not equal 100% due to rounding

### Use of Mobile Devices

Differences in participant reports of behavior in accessing information away from and during WIL were found for several variables (Table 2). Activities were categorized into work, non-work, and study-related tasks. Work-related activities were patient-centered activities that occurred at point of care, or related to education or professional development. Non-work related activities involved communication and personal tasks that were not of the nature or scope required in the workplace.

### Non-Work Related Activities

Differences in access to information using a mobile or portable device away from or at the workplace, were reported for 6 out of the 7 items grouped in non-work related activities. Non-work related uses of portable devices were more frequent when students were away from the workplace. These included messaging (median 4 vs median 2, *T*=49.5, *P*<.001), social networking (median 4 vs median 1, *T*=48.5, *P*=.01), shopping on the Internet (median 2 vs median 1, *T*=17.5, *P*=.01), conducting personal business online (median 2 vs median 1, *T*=48.0, *P*=.01), and checking or sending non-work related texts or emails to co-workers (median 2 vs median 1, *T*=43.0, *P*=.04).

### Study-Related Activities

Study-related activities that were conducted more regularly away from the workplace included browsing on the Internet (median 4 vs median 3, *T*=16.5, *P*<.001), or accessing University sites for information (median 4 vs median 3, *T*=63.0, *P*=.01). Checking or sending study-related text messages or emails to friends or co-workers also occurred (median 3 vs median 2, *T*=43, *P*=.01).

### Away From Work-Integrated Learning

There were no differences found away from or during WIL for accessing work-related activities such as accessing drug, nursing, and medical information or professional education and development resources. Students reported infrequently accessing study-related text or email messages from academic supervisors, or submitting assessment tasks using a mobile or portable device. Respondents also used a mobile or portable device as a clock or a stopwatch (median 4 vs median 2, *T*=61.5, *P*=.01) more regularly away from the workplace.

### During Work-Integrated Learning

Participants reported that during WIL they did not shop on the Internet; check or post on social networking sites; play online or games loaded on the device; conduct personal business online; or check/send personal text messages or emails to co-workers. Access to work-related protocols and mobile apps that assist with patient or client care were more likely (once per day) to be accessed during WIL.

### Non-Access

Respondents reported they did not access sites for patient handouts and teaching, communicating with other members of the health care team to coordinate patient or client care, or to play games.

**Table 2:** Utilization of portable or mobile devices during work integrated learning.

Use of portable or mobile devices to access information		Away from PEP Median, (Range)	During PEP Median, (Range)	*P* value
**Work-related activities**				
	I access work-related drug references	3 (1-5)	3 (1-5)	.94
	I use it to communicate with other members of the health care team to coordinate patient or client care	1 (1-5)	1 (1-5)	.94
	I access work-related protocols	1 (1-5)	2 (1-5)	.90
	I access work-related apps that assist patient or client care	1 (1-5)	2 (1-5)	.75
	I access sites for patient handouts and teaching	1 (1-5)	1 (1-5)	.53
	I use the device as a calculator for nursing/medical formulas	2 (1-5)	2 (1-5)	.52
	I access sites for professional education and development	3 (1-5)	3 (1-5)	.23
	I access work-related nursing/medical information	3 (1-5)	3 (1-5)	.21
**Non-work related activities**				
	I check/send personal text messages or emails to family or friends	4 (1-5)	2 (1-5)	<.001
	I check/post on social networking sites (Facebook, Twitter, Snapchat etc)	4 (1-5)	1 (1-5)	.01
	I shop on the Internet	2, (1-5)	1 (1-5)	.01
	I conduct personal business online (eg paying bills, banking)	2 (1-5)	1 (1-5)	.01
	I play games loaded on the device^a^	1 (1-5)	1 (1-5)	.05
	I check/send personal text messages or emails to co-workers	2 (1-5)	1 (1-5)	.04
	I play online games	1 (1-5)	1 (1-5)	.84
**Study-related activities**				
	I browse (eg use a search engine Google, Safari etc) for information to assist with progression of my studies^a^	4 (1-5)	3 (1-5)	< .001
	I check/send study related text messages or emails to friends or co-workers	3 (1-5)	2 (1-5)	.01
	I access University related sites (eg MyLO) to assist with progression of my studies^a^	4, (1-5)	3, (1-5)	.01
	I check/send study related text messages or emails to my academic supervisors^a^	2, (1-5)	2, (1-5)	.26
	I access study related sites (eg library, journal articles) to assist with progression of my studies^a^	3, (1-5)	2, (1-5)	.03
	I submit assessment tasks^a^	2, (1-5)	2, (1-5)	.44
**Other activity**				
	I use the device as a clock or stopwatch^a^	4, (1-5)	2, (1-5)	.01

^a^Non-validated question.

## Discussion

### Principal Findings

This study demonstrated differences in accessing the Internet or device-based resources using a mobile or portable device at, and away, from the workplace by undergraduate nurses. Undergraduate nurses reported there was a range of non-work related Internet-based activities they avoided during WIL. Predominantly these activities related to social networking with family or friends, shopping, or conducting personal business online. McBride, Le Vasseur, and Li [6] and others [4, 7,20,32] indicated that risks to patient or client safety could be attributed to individual level distraction at point of care. While distraction may occur while using a mobile or portable device during WIL [4,6], this study found it was unlikely due to student nurses’ accessing non-work related sites.

The research indicates that through lack of access to mobile devices or resources there were lost opportunities to engage with patients or clients at point of care. Undergraduate nurses reported they never accessed patient handouts for teaching or communicating with other members of the health care team to coordinate patient care. At registration there is an expectation that students are work-ready [38,39]. There is an expectation that students will develop professional identity during their course and during their final year, during WIL, they will develop the knowledge, skills, attitudes, and behavior that demonstrate competency for registration [10]. A key role for nurses is providing patients with health education, and with guidance, final year students may initiate and engage patients in improving their health literacy. A lack of access to web-based resources at point of care can hinder or undermine this development of professional identity [9,10]. Additionally, senior undergraduate students could be involved with coordination of patient care if they had the opportunity. Self-management education at point of care creates opportunity for shared understandings that can improve health outcomes of patients or clients by enabling access to resources and individualized treatment plans [28,34]. Modeling of professional behaviors required as a graduate nurse, including access to web-based self-management or health education resources, could promote work-readiness of students and minimize transition shock [38,39].

There was no demonstrated difference in behavior for accessing work-related drug references, nursing or medical information, and professional education and development. Undergraduate students continue to study when they are not at the workplace. The convenience of enabling access to mobile or portable devices *in situ* could promote habits that support continuing professional development and life-long learning which are requirements for continuing registration [40,41].

Differences were found in browsing for information, accessing study or University related sites, which predominantly occurred away from WIL. The convenience and ease of using a mobile device supported student-centered learning [2,41] away from and during WIL. Although no differences were found, mobile devices were used for contact with academic supervisors and submission of assessments. Access to mobile devices enables the activity of learning to be user-controlled [2]. The convenience and ease of learning in real-time at point of care challenges traditional pedagogy. Utilization of mobile devices to access a range of study information has implications for learning at systems, organizational and individual levels that need to be acknowledged and addressed through curriculum design and organizational policy. Addressing educational preparation in ICT competency and guidance in safe and appropriate use of mobile learning in the classroom, prior to undertaking WIL, could assist with the development of professional identity. Policy development to guide undergraduate students and health profession staff about effective and competent use of mobile devices *in situ* could also ameliorate the risk of distraction at point of care.

While away from the workplace students tend to use mobile or portable devices to monitor time. This behavior was less likely during WIL, suggesting that undergraduate students did not access their mobile device to conduct patient observations such as pulse or respiration assessments. Institution or organization policy that dissuades the use of mobile or portable devices during WIL may be a factor for regulating use [33]. Concerns about cross infection between patients could also prohibit the use of a mobile device for this intimate patient activity [36,37].

Respondents indicated that communicating with family, friends, co-workers, and study were more likely to be accessed than playing online games loaded on the device while away from or during WIL. Communication or maintenance of meaningful relationships may contribute to lack of interest in playing games using a mobile device. The predominance of females in the cohort may also have negatively skewed the result as females are less likely than males to game [42].

### Limitations

This study had several limitations. The first included the low response rate. This may have occurred because although the survey was anonymous, it may have contributed to students feeling that if they did not respond appropriately there was a chance of disadvantage with their studies. Additionally, survey fatigue of students may also have contributed to a lower level of engagement with completion of the survey. Respondents were recruited from one university and may attend WIL at partner health care organizations that have guidelines impacting the conduct by students during WIL, which could reduce the generalizability of the findings. Of the questions asked, 7 relating to access to study options were not validated. In these cases the sentence construction was similar to the validated questions, however their actual reliability is unknown at this time. Finally, as this survey has been administered by staff at the teaching university there is the possibility of social desirability bias, the tendency to respond to questions in a known socially acceptable manner.

### Future Directions

Further examination of preferred mobile or portable devices used for L&T by undergraduate nurses is warranted. Review of higher education institutional and health care organization policy relating to mobile devices could reveal there is a need to change to allow students to prepare for their future profession in accessing learning objects or resources while they are undertaking WIL. Concurrently, there is a need to ensure ICT architecture and infrastructure at organizations supports L&T at the workplace. Curriculum design to incorporate appropriate and safe use of mobile devices is necessary to promote diffusion of this informal method of L&T into the workplace. Over time, responsible use of mobile devices to minimize risk could create a cultural shift that will enable safe use for L&T *in situ* at point of care.

### Conclusions

Exploration of access to information using a mobile or portable device by undergraduate nurses away from and during WIL contributes to the discourse about the challenges of using these devices at systems, organizational, and individual levels. This study found that undergraduate nurses limited their access to non-work or non-patient-centered care while undertaking WIL. Furthermore, the risk of distraction was unlikely due to student nurses’ accessing non-work related sites (4,6). The use of mobile devices for study purposes occurred during WIL, but was more frequent away from the workplace. This suggests students were focused on developing competency in patient care while in the workplace. Acceptance of access to mobile devices as a legitimate L&T tool during WIL is imperative. To support this aim there is a need to promote professional identity and facilitate L&T by including guidance for appropriate mobile learning behavior in the curriculum. The development of best practice guidelines or policy to minimize risk and enable improvement of health outcomes of patients at point of care is necessary. Undergraduate students are the next generation of nurses. This study showed they can discern appropriate mobile device use. Over time, nurses will expect easy access to mobile learning resources to enable them to deliver safe and effective health care to patients.

## Abbreviations

ICT: information communication technology
L&T: learning and teaching
mLearning: mobile learning
WIL: work-integrated learning
